# What drives the shift between sexual and clonal reproduction of *Caragana stenophylla* along a climatic aridity gradient?

**DOI:** 10.1186/s12870-018-1313-6

**Published:** 2018-05-22

**Authors:** Zhongwu Wang, Lina Xie, Chelse M. Prather, Hongyu Guo, Guodong Han, Chengcang Ma

**Affiliations:** 1 College of Grassland, Resources and Environment, Inner Mongolia Agricultural University, Hohhot, 010018 China; 2 Tianjin Key Laboratory of Animal and Plant Resistance, College of Life Sciences, Tianjin Normal University, Tianjin, 300387 China; 3 College of Life Sciences, Nankai University, Tianjin, 300071 China; 4 Department of Biology, University of Dayton, Dayton, OH 45419 USA

**Keywords:** Clonal plant, Priority of sexual reproduction, Reproductive cost, Reproductive mode, Climate

## Abstract

**Background:**

The reasons that clonal plants shift between sexual and clonal reproduction have persisted as a knowledge gap in ecological literature. We hypothesized that clonal plants’ shifts between sexual and clonal reproduction in different environments are driven by the relative costs of sexual and clonal reproduction. Moreover, we hypothesized plants prioritize sexual reproduction over clonal reproduction. To test these hypotheses, we determined the costs of sexual and clonal reproduction, and proportions of sexual and clonal reproduction of *Caragana stenophylla* along a climatic aridity gradient (semi-arid, arid, very arid and intensively arid zones) in the Inner Mongolia Steppe using several complementary field experiments.

**Results:**

The cost of sexual reproduction increased while the cost of clonal reproduction decreased as climatic drought stress increased from the semi-arid to the intensively arid zones. The changes in the costs of these reproductive modes drove a shift in the reproductive mode of *C. stenophylla* from more sexual reproduction in the semi-arid zone to more clonal propagation in the intensively arid zone. However, because of the evolutionary advantages of sexual reproduction, sexual reproduction still held priority over clonal production in *C. stenophylla*, with the priority of sexual reproduction gradually increasing from the semi-arid to the intensively arid zones.

**Conclusions:**

Our study suggested that sexual reproduction has relatively high priority in propagation of *C. stenophylla*. However, if the costs of sexual reproduction are too high, *C. stenophylla* likely chooses clonal reproduction, and the ratio between sexual and clonal reproduction could be mediated by reproductive cost. These reproductive strategies reflect optimal resource utilization, and allow the persistence of both reproductive modes across stressful conditions depending on their evolutionary advantages.

## Background

For majority of organisms, sexual reproduction is often considered essential for evolutionary adaptation by producing genetically variable offspring [1]. The advantages of sexual reproduction include increasing offspring diversity [2], reducing mutational load [3], increasing adaptive potential to environmental variation and disturbances [4–6], and greater dispersal potential for seeds compared with ramets [2, 4, 6]. However, sexual reproduction often requires more energy [7–9], more time [9], and has lower efficiency compared with clonal reproduction [10]. The number of sexually produced offspring may be limited by the availability of mates [1]. Sexual reproduction, therefore, is often recognized as a more costly reproduction strategy that has evolutionary advantages [11, 12]. On the other hand, clonal reproduction (resprouting) limits genetic variation and recombination opportunities for adaptive evolution, which may accumulate deleterious mutation loads leaving the population vulnerable to environmental changes [12]. Consequently, clonal reproduction offers increased reproductive assurance when sexual reproduction is difficult [1, 12–15] and avoids the relatively high costs of sexual reproduction [2, 12]. Therefore, clonal reproduction is often more important for population growth than sexual reproduction in clonal plants [6]. No study has explicitly compared between the costs of sexual and clonal reproduction, and the relative costs of these strategies may be context dependent.

When sexual and clonal reproduction can occur simultaneously for individual plants, the organism must allocate resources among the seeds and new ramets, thereby resulting in a trade-off between the two reproductive modes [6, 12, 16, 17]. Abiotic factors and biotic factors can shift the trade-offs between sexual and clonal reproduction [13]. Plants tend to choose sexual reproduction when experiencing: climatic conditions favorable to plant growth [18–20], higher altitudes [21, 22], years with conditions favorable to plants [23]. Additionally, those plants that have an annual life-cycle [24], or are older populations [22] are also more likely to choose sexual reproduction. By contrast, plants tend to choose clonal reproduction under harsh climate conditions [18], after disturbances [2, 11, 25], at low altitudes [22, 26], in weedy habitats [27], in unfavorable years [23], in habitats with plentiful resources [28], in species with perennial life-cycle [24], and in younger populations [22]. Plants with the capability of altering their mode of reproduction depending on environmental conditions may thrive in a broader range of conditions [9] and be more resilient in a longer term [29].

It is commonly thought that sexual reproduction is evolutionary reproductive mode [1], and that clonal reproduction occurs because of obstacles to sexual reproduction caused by genetic or environmental factors [4, 12, 14, 30–33], such as male sterility [34], mate limitation [35, 36], low rates of fertilization, or embryo/fruit abortion [37]. However, the mechanisms for plants to shift between sexual and clonal reproduction under different environmental conditions have not been explicitly examined [4, 6, 14], and we are aware of no studies that have examined the reproductive costs of plants choosing reproductive modes.

Generally, selections of many biological traits are a result of the need to conserve resources. For example, leaf deciduousness reduces resource consumption, thin leaf blades for save the expense of building structure material, and shrunken stomata decrease conserve water otherwise lost through transpiration [38]. Some critical stages of sexual reproduction, such as flowering [33], fruit setting [12, 39, 40], seed field preservation [41] and seedling establishment [12, 20, 42] make a plant more susceptible to abiotic and biotic stresses. Abortive reproduction processes lead to the wasteful investment and raise the cost of reproduction. Conversely, most clonal processes occur belowground where soils can buffer the effects of environmental stressors, thus clonal reproduction is less affected by the environment [12, 15, 20]. Costs of sexual reproduction may be more environmentally dependent than that of clonal reproduction. The different environmental dependencies between costs of sexual and clonal reproductive modes may change the rates at which plants use the two reproductive modes along environmental gradients. If these different reproductive modes have indeed evolved to save resources, we hypothesized that shifts between these reproductive modes in different environments is driven by the relative costs of sexual and clonal reproduction. In other words, plants should choose the most economic balance between the two reproductive modes to achieve high efficiency of resource use. Additionally, because sexual reproduction has many evolutionary advantages, plants should allow higher costs to maintain their sexual reproduction for these benefits. Therefore, we hypothesized that clonal plants prioritize sexual versus asexual reproduction if the cost is affordable.

The clonal plant, *Caragana stenophylla* Pojark, is the main shrub species in Inner Mongolia Steppe with a distribution ranging from the semi-arid zone to the intensively arid zone [18, 43]. It is both economically valued as fodder, green manure, and a honey resource, and environmentally valued for the attenuation of wind erosion, sand fixation, water and soil conservation, as well as other important roles in mediating grassland ecosystem functions and services [39]. *C. stenophylla* sexually reproduces by establishing saplings through seed germination and clonally reproduces by establishing ramets from adventitious buds on horizontal roots [44]. We previously showed that the proportions of sexual reproduction decreased and the clonal reproduction increased with increasing drought stress from the semi-arid zone to the intensively arid zone [18]. The underlying factors that drive the shift between sexual and clonal reproduction of *C. stenophylla* along this climatic aridity gradient, however, remain unclear.

To test our hypotheses on the mechanisms for plants to shift between sexual and clonal reproduction, we examined the costs of sexual and clonal reproduction, and proportion of sexual and clonal reproduction for *C. stenophylla* populations along a climatic aridity gradient (semi-arid zone, arid zone, very arid and intensively arid zones) in the Inner Mongolia Steppe, analyzed relationship between reproductive proportions and reproductive costs across this gradient. Our study provides important insight into the underlying mechanisms driving clonal plants’ shift between sexual and clonal reproduction under different environmental conditions.

## Methods

### Study sites

We conducted this study in the Inner Mongolian Steppe in northern China that has a strong gradient of climatic aridity from the northeast to the southwest, along which there are semi-arid, arid, very arid, intensively arid, and extremely arid zones. Therefore, it provides an ideal system to explore the variations in reproduction strategies of a plant species along a climatic aridity gradient. We worked at four study sites: (1) Xilinhaote in the semi-arid zone, (2) Siziwang in the arid zone, (3) Etuoke in the very arid zone and (4) Alashanzuo in the intensively arid zone in Inner Mongolia Steppe (Table 1).

**Table 1:** Location and environmental data of the four field sites in the Inner Mongolia Steppe

Site	Longitude (°E)	Latitude (°N)	Altitude (m)	Mean annual precipitation (mm)	Annual mean temperature (°C)	Sunshine duration (h/year)	Aridity index (AI)	Moisture types
Xilinhaote	115°55′19”	44°28′31”	990	281	2.35	2932	0.174	Semi-arid
Siziwang	111°53′22”	41°47′28”	1492	240	3.40	3065	0.128	Arid
Etuoke	107°58′02”	39°07′02”	1500	210	6.40	3050	0.070	Very arid
Alashanzuo	105°41′34”	38°19′47”	1561	110	7.80	3200	0.034	Intensively arid

Aridity index (AI) = precipitation / potential evapotranspiration [52]

As severe grazing would significantly limit sexual reproduction [42, 45], we carried out the study only in non-grazed and mildly grazed grassland. Within each study site, we established four ~ 3 ha plots (two non-grazed plots and two mildly grazed plots). Since vegetation cover decreases as drought stress increases from the semi-arid zone to the intensively arid zone, the number of animals grazing the mildly grazed plots at each site was set according to the amount of plant biomass (i.e., food) available. The overall grazing intensity at each study site was: 1.2 sheep per hectare in the semi-arid zone, 1.0 sheep/ha in the arid zone, 0.8 sheep/ha in the very arid zone, and 0.4 sheep/ha in the intensively arid zone. All surveys were conducted twice in the two successive years.

### Cost of sexual reproduction

In this study, cost of sexual reproduction was valuated using the gross biomass cost for establishing one sapling.

We determined the cost of seed production in the flowering season of 2012 and 2013. In each plot and each year, we established a transect of 100 m, selected 10 *C. stenophylla* shrub clusters at intervals of 10 m along the transect (i.e., in total 20 shrub clusters per plot in the two years). For each shrub cluster, we marked a representative vigorous sampling branch whose flowering rate would be representative of the shrub cluster. Every 3 days, we counted both the total number of flowers and the numbers of young pods and mature pods per branch. After all pods matured, we harvested all mature pods and counted the number of seeds in each. Additionally, we randomly collected 100 flowers, 50 young pods, and 50 mature pods from at least 20 shrub clusters in each plot annually. We dried (80 °C for 72 h) and weighted biomass, and calculated average flower, young pod, and mature pod biomass for each plot, respectively.

We calculated flowering efficiency as the number of seeds per bloom in each plot.

$$ Flowering\ efficiency\ \left( seed/ flower\right)= number\ of\ seeds/ number\ of\ flowers $$

We calculated the cost of seed production (cost of seed production is defined as the biomass expenditure of shrub for producing one seed) in each plot. In the intensively arid zone, there were no mature pods on some of the sampling branches, thus we could not calculate cost of seed production with sampling shrubs as random variables.

$$ Cost\ of\ seed\ production\ \left(g\  biomass/ seed\right)=\left[ average\ flower\ biomass\times number\ of\ flower s+ average\ young\  pod\  biomass\times \left( number\ of\ young\ pods- number\ of\ mature\ pods\right)+ average\ mature\  pod\  biomass\times number\ of\ mature\ pods\right]/ seed\ number $$

Annually during June–July, we collected and air-dried healthy *C. stenophylla* seeds from each of the four sites respectively. We conducted the field experiments for sapling establishment rate in 2013 and 2014, respectively. At the beginning of each growing season, we sowed seeds collected from the same site in the previous year in each plot. In each year, we established a transect of 90 m in each plot, and we placed nine quadrats (1 m × 1 m) at intervals of 10 m along the transect, and thus we sampled in total 18 quadrats for each plot within the two years. The quadrats were under/in shrubs, grasses or open areas. We sowed 100 seeds in each quadrat. Our previous study has investigated the seed densities of *C. stenophylla* in soils inside shrub canopies (semi-arid zone: 4 seeds/m^2^; arid zone: 3 seeds/m^2^; very arid zone: 2 seeds/m^2^; intensively arid zone: 1 seed/m^2^) and outside shrub canopies (semi-arid zone: 3 seeds/m^2^; arid zone:1 seed/m^2^; very arid zone: 1 seed/m^2^; intensively arid zone: 0 seed/m^2^) [43]. Thus, we deducted the number of pre-existing seeds in soil when sowing seeds. At 18 months after sowing (at the end of the growing season in the second year), we recorded number of saplings in each quadrat, and then calculated sapling establishment rate (sapling number / seed number) in each quadrat and each plot. Based on cost of seed production and sapling establishment rate, we calculated cost of sexual reproduction (gross biomass cost for establishing one sapling) for each plot according to the following formula.

$$ Cost\ of\ sexual\ reproduction\ \left( CSR;g\  biomass/ sapling\right)= cost\ of\ seed\ production/ sapling\ establishment\ rate $$

### Cost of clonal reproduction

In this study, cost of clonal reproduction was valuated using the gross biomass cost for establishing one ramet. Clonal reproduction of *C. stenophylla* produces 20 cm–130 cm horizontal roots (spacers) from taproots of the mother plant or adult ramets, then at the end or the middle of the horizontal roots, vertical roots grow deeper and adventitious buds grow towards soil surface. Finally, the adventitious buds emerge aboveground, forming a new ramet. Spacers usually do not break after the ramet establishment.

We surveyed the cost of clonal reproduction in 2013 and 2014, respectively. In each year, we randomly sampled 10 *C. stenophylla* shrub clusters in each plot, and thus we sampled in total 20 shrub clusters per plot in the two years. For each shrub cluster sampled, we first carefully removed the sand around each cluster (if any was there), and then we dug down 30–40 cm from the surface to expose the underground parts of all plants, including mother plant, ramets and new sprouting ramets (1.0–3.0 m^2^). We measured all spacer lengths, and calculated the mean spacer length for each shrub cluster. We cut off all new sprouting ramets from the base of the mother plant (or the adult ramet) and measured dry biomasses of each new sprouting ramets (dried at 80 °C for 72 h), then calculated the mean dry biomasses of one new sprouting ramet for each shrub cluster and for each plot. The dry biomasses of one new sprouting ramet represented the cost for formation one ramet.

Physical connections with the adult plant improved fitness of these new sprouting ramets, thus majority new sprouting ramets could establish, but a few of them died. We surveyed the survival rate of newly sprouting ramet at 18-month in 2013 and 2014, respectively. In spring of each year, we marked 50 new sprouting ramets in each plot (100 new sprouting ramets per plot in the two years). At the end of the growing season in the next year, we recorded the number of survival ramets among the marked ones, then calculated newly sprouting ramet survival rate for each plot.

Based on the cost for formation one ramet and the newly sprouting ramet survival rate in each plot, we calculated cost of clonal reproduction (gross biomass cost for establishing of one ramet) for each plot according to the following formula.

$$ Cost\ of\ clonal\ reproduction\ \left( CCR;g\  biomass/ ramet\right)= cost\ for\ for mation\  one\  ramet/ newly\ sprouting\ ramet\ survival\ rate $$

We calculated the reproductive cost ratio for each plot according to the following formula.

$$ Reproductive\ cost\ ratio= CCR/ CSR $$

### Reproduction ratio

We surveyed of reproduction ratio in 2013 and 2014, respectively. In each year, we randomly sampled 10 *C. stenophylla* shrub clusters in each plot (20 shrub clusters per plot in the two years). For each shrub cluster sampled, we dug down 30–40 cm from the surface to expose the underground parts of all plants, including mother plant and ramets (1.0–3.0 m^2^). We recorded the number of seed-derived offspring and the number of sprouting-derived offspring. We calculated the proportion of sexual and clonal reproduction for each shrub cluster respectively according to the following formulas.

$$ Proportion\ of\ sexual\ reproduction=\left[ seed- derived\ of fspring\ number/\left( seed- derived\ of fspring\ number+ sprouting- derived\ of fspring\ number\right)\right]\times 100\% $$

$$ Proportion\ of\ clonal\ reproduction=\left[ sprouting- derived\ of fspring\ number/\left( seed- derived\ of fspring\ number+ sprouting- derived\ of fspring\ number\right)\right]\times 100\% $$

Then we calculated the reproduction ratio for each plot according to the following formula.

$$ Reproduction\ ratio= number\ of\ sexual\ reproduction/ number\ of\ clonal\ reproduction $$

### Sexual reproduction priority

An economical reproductive strategy is that the lower the cost of one reproductive mode is, the larger the proportion of individuals in a population should be using that mode. If reproduction ratio of *C. stenophylla* was entirely mediated by reproductive cost, ratio of sexual versus clonal reproduction would be equal to ratio of CCR versus CSR. While, if ratio of sexual versus clonal reproduction is higher or lower than ratio of CCR versus CSR, it would indicate that reproductive mode choosing of *C. stenophylla* is not entirely mediated by reproductive cost, and sexual reproduction or clonal reproduction is prior between the two reproductive modes.

We quantified sexual reproduction priority using priority index, and sexual reproduction priority index (SRPI) was calculated for each plot according to the following formula.

$$ SRPI=\left( reproduction\ ratio- reproductive\ cost\ ratio\right)/ reproductive\ cost\ ratio $$

Positive SRPI values indicate that sexual reproduction has priority; negative SRPI values indicate that clonal reproduction has priority; SRPI of zero (or indistinguishable from zero) indicate neither mode takes priority over another.

### Statistical analyses

We performed analyses using GLMMs with sampling shrubs within each plot and plots within each climate zone as random variables (sampling shrubs (20 shrubs per plot) were nested in plot; plots (4 plots per climate zone) were nested in climate zone) to examine the differences of spacer length, cost for formation one ramet, and proportion of sexual reproduction and clonal reproduction among climate zones. Similarly, we performed analyses using GLMMs with quadrats within each plot and plots within each climate zone as random variables (quadrats (18 quadrats per plot) were nested in plot; plots (4 plots per climate zone) were nested in climate zone) to examine the differences of sapling establishment rate among climate zones. We performed analyses using GLMMs with plots within each climate zone as random variables (plots (4 plots per climate zone) were nested in climate zone) to examine the differences of flowering efficiency, biomass cost of seed production, CSR, newly sprouting ramet survival rate, CCR, and SRPI among climate zones, and differences between CSR and CCR in the same climate zone. We performed regression analyses to examine the changes of reproductive cost ratio (CCR / CSR) and reproduction ratio (sexual reproduction/ clonal reproduction) along the climatic aridity gradient, and exponential curve has the highest correlation coefficient. All data analyses were performed with SPSS 21.0 (SPSS Inc).

## Results

### Cost of sexual reproduction and clonal reproduction

Seed production is the first important stage of sexual reproduction. Under the relatively benign climatic conditions of the semi-arid zone, *C. stenophylla* produced fewer but larger flowers, and relatively higher flowering efficiency (~ 0.846 seed/ flower). In contrast, under the harsher climatic conditions of the intensively arid zone, *C. stenophylla* produced more but smaller flowers, and lower flowering efficiency (~ 0.021 seed/ flower). While in the arid and very arid zones, *C. stenophylla* exhibited intermediate flowering efficiency (arid zone ~ 0.551 seed/ flower; very arid zone ~ 0.363 seed/ flower) between the semi-arid and intensively arid zones (Table 2). Alterations in flowering efficiency led to a substantial increase in cost of seed production from the semi-arid zone to the intensively arid zone (Table 2). Establishment of sapling from seed is another important stage of sexual reproduction. Sapling establishment rate sharply decreased from the semi-arid zone (~ 0.681 × 10^− 2^ sapling/ seed) to the intensively arid zone (~ 0.097 × 10^− 2^ sapling/ seed, Table 2). Substantial increases in the cost of seed production together with sharp decrease in sapling establishment rate resulted in drastically increases in the cost of sexual reproduction (CSR) of *C. stenophylla* from the semi-arid zone to the intensively arid zone. Compared to the CSR in the semi-arid zone, CSR increased 3.08 times in the arid zone, 5.81 times in the very arid zone, and 100.67 times in the intensively arid zone, respectively (Table 2). Our results suggested that CSR was greatly influenced by the climatic aridity gradient.

**Table 2:** Cost of sexual reproduction of *C. stenophylla* in the four climatic aridity zones (mean ± SE)

	Semi-arid zone	Arid zone	Very arid zone	Intensively arid zone	GLMM test results
Flowering efficiency (seeds/flower)	0.846 ± 0.034	0.551 ± 0.048	0.363 ± 0.026	0.021 ± 0.001	F_3,12_ = 50.07, *P* < 0.01
Cost of seed production (mg biomass/seed)	36.12 ± 0.87	48.05 ± 3.22	55.64 ± 2.37	494.35 ± 16.13	F_3,12_ = 312.83, *P* < 0.01
Sapling establishment rate (sapling /seed) × 10^-2^	0.681 ± 0.076	0.292 ± 0.054	0.181 ± 0.046	0.097 ± 0.035	F_3,284_ = 3.93, *P* < 0.01
CSR (g biomass/sapling)	5.35 ± 0.33	16.48 ± 0.86	31.06 ± 1.27	546.54 ± 89.09	F_3,12_ = 13.67, *P* < 0.01

*CSR* cost of sexual reproduction

The spacer length of *C. stenophylla* decreased from the semi-arid zone (63.9 cm) to the intensively arid zone (20.3 cm). In contrast, the spacer diameter increased from the semi-arid zone to the intensively arid zone. Therefore, the cost of the formation of one ramet decreased from the semi-arid zone (7.03 g) to the intensively arid zone (4.26 g). Additionally, ramet survival rate decreased from the semi-arid zone (91.0%) to the intensively arid zone (71.0%). These variations led to a slight decrease in the cost of clonal reproduction (CCR) of *C. stenophylla* from the semi-arid zone to the intensively arid zone. However, the CCR in the semi-arid zone was only 1.05 times as high as that in the arid zone, 1.19 times as high as that in the very arid zone, and 1.28 times as high as that in the intensively arid zone, respectively (Table 3). These results suggest that CCR might not be strongly influenced by the climatic aridity gradient.

**Table 3:** Cost of clonal reproduction of *C. stenophylla* in the four climatic aridity zones (mean ± SE)

	Semi-arid zone	Arid zone	Very arid zone	Intensively arid zone	GLMM test results
Spacer length (cm)	63.9 ± 2.8	40.9 ± 2.4	25.5 ± 0.6	20.3 ± 0.5	F_3,316_ = 3.45, *P* < 0.05
Cost for formation one ramet (g biomass/ramet)	7.03 ± 0.31	6.14 ± 0.36	4.84 ± 0.12	4.26 ± 0.11	F_3,316_ = 0.77, *P* = 0.514
Newly sprouting ramet survival rate (%)	91.0 ± 2.6	82.5 ± 2.4	75.0 ± 3.1	71.0 ± 3.7	F_3,12_ = 3.73, *P* < 0.05
CCR (g biomass/ramet)	7.74 ± 0.42	7.37 ± 0.23	6.50 ± 0.45	6.04 ± 0.34	F_3,12_ = 1.95 *P* = 0.175

*CCR* cost of clonal reproduction

### Relationship between reproductive cost and reproduction ratio

In the semi-arid zone, CSR of *C. stenophylla* was less than CCR; but in the arid, very arid and intensively arid zones, CSRs were higher than CCRs. In the intensively arid zone, CSR was 89.17 times higher than CCR (Table 4). Reproductive cost ratio (CCR / CSR) of *C. stenophylla* exponentially decreased from the semi-arid zone to the intensively arid zone (Table 4). The proportion of those using sexual reproduction decreased but the proportion of those plants clonal reproduction increased from the semi-arid zone to the intensively arid zone, thus reproduction ratio (sexual reproduction/ clonal reproduction) also exponentially decreased from the semi-arid zone to the intensively arid zone (Table 4). These results indicated that there likely was a negative relationship between reproductive cost and reproduction proportion, and that for a specific reproductive mode, a lower reproductive cost corresponds to larger proportion of plant using that mode of reproduction.

**Table 4:** Reproductive cost ratio and reproduction ratio of *C. stenophylla* in the four climatic aridity zones (mean ± SE)

	Semi-arid zone	Arid zone	Very arid zone	Intensively arid zone	Test method and result
CSR (g biomass/sapling)	5.35 ± 0.33	16.48 ± 0.86	31.06 ± 1.27	546.54 ± 89.09	–
CCR (g biomass/ramet)	7.74 ± 0.42	7.37 ± 0.23	6.50 ± 0.45	6.04 ± 0.34	–
GLMM test result of differences between CSR and CCR	F_1,6_ = 8.488 *P* < 0.05	F_1,6_ = 52.162 *P* < 0.01	F_1,6_ = 146.593 *P* < 0.01	F_1,6_ = 14.519 *P* < 0.01	–
Reproductive cost ratio (CCR / CSR)	1.47 ± 0.16	0.45 ± 0.02	0.21 ± 0.02	0.01 ± 0.00	Regression analysis, *R*^2^ = 0.906, *P* < 0.01
Proportion of sexual reproduction	67.3%	40.0%	27.5%	10.7%	GLMM F_3,316_ = 73.51 *P* < 0.01
Proportion of clonal reproduction	32.7%	60.0%	72.5%	89.3%	
Reproduction ratio (sexual reproduction/ clonal reproduction)	2.08 ± 0.15	0.67 ± 0.04	0.38 ± 0.02	0.12 ± 0.01	Regression analysis, *R*^2^ = 0.967, *P* < 0.01

*CSR* cost of sexual reproduction, *CCR* cost of clonal reproduction

In addition, reproduction ratio (sexual reproduction/ clonal reproduction) of *C. stenophylla* were higher than ratios of reproductive cost ratio (CCR / CSR) in all the four climate zones (Table 4).

### Sexual reproduction priority

**Fig. 1:**
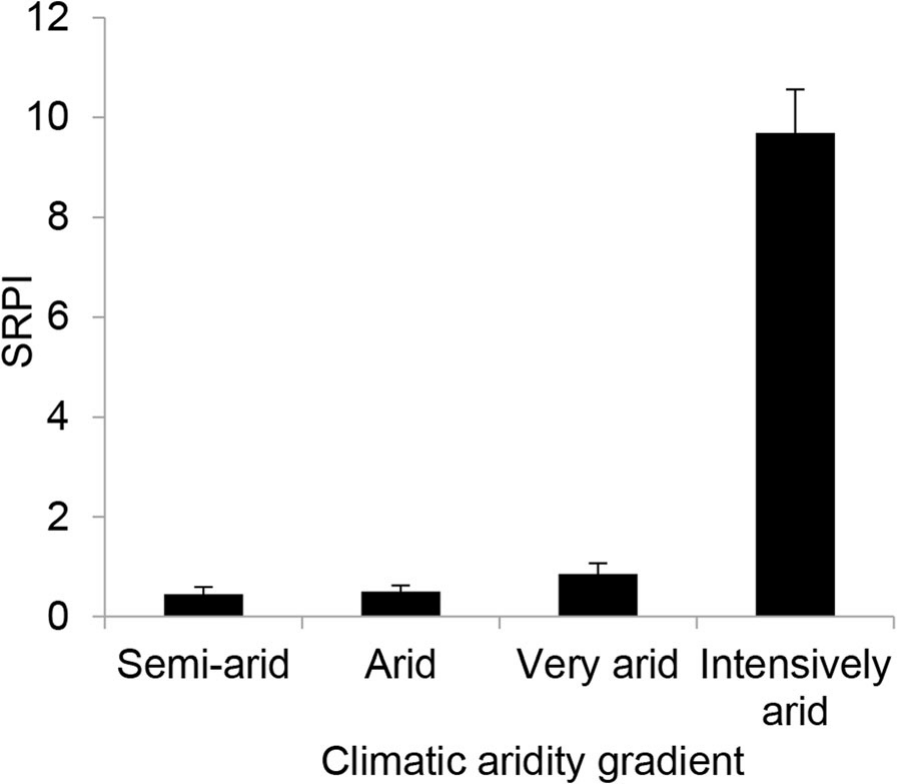
Sexual reproduction priority index (SRPI) of *C. stenophylla* in the four climatic aridity zones. GLMM result, F_3,12_ = 98.170, *P <* 0.01

If reproduction proportion of *C. stenophylla* was entirely mediated by reproductive cost, the optimal proportion (predicted based on reproductive cost) of sexual versus clonal reproduction would be 59.5% sexual reproduction versus 40.5% clonal reproduction in the semi-arid zone, 31.0% versus 69.0% in the arid zone, 17.4% versus 82.6% in the very arid zone, and 1.0% versus 99.0% in the intensively arid zone, respectively. However, the actual reproductive proportions were 67.3% sexual reproduction versus 32.7% clonal reproduction in the semi-arid zone, 40.0% versus 60.0% in the arid zone, 27.5% versus 72.5% in the very arid zone, and 10.7% versus 89.3% in the intensively arid zone, respectively (Table 4). The actual proportions of sexual reproduction were higher than the optimal proportion of sexual reproduction in all the four climate zones. These results showed that sexual reproduction is prior for *C. stenophylla* in choosing reproductive modes. Sexual reproduction priority indexes (SRPI) of *C. stenophylla* were positive in all the four zones. SRPIs gradually increased from the semi-arid zone to the intensively arid zone (Fig. 1).

## Discussion

### Reproductive cost drives the shift of reproduction modes

Our results suggested that cost of sexual reproduction (CSR) was greatly influenced by the climatic aridity gradient, but cost of clonal reproduction (CCR) was not significantly influenced by the climatic aridity gradient. The sharp increase in CSR and slight decrease in CCR led to a great change in relative balance between CSR and CCR as climatic drought stress increased from the semi-arid zone to the intensively arid zone (Table 4).

The CSR of *C. stenophylla* was lower than CCR in the semi-arid zone. Sexual reproduction not only was economical when environmental stressors were limited, but also could increase offspring diversity [2] and may confer the ability to adapt to environmental variation and disturbances [4–6]. Therefore, *C. stenophylla* population in the semi-arid zone has more seed-derived offspring (67.3%, Table 4). Another advantage of sexual reproduction is that it can provide greater dispersal potential [2, 4, 6]. The *C. stenophylla* population in the semi-arid zone was mainly recruited by sexual reproduction, thus it exhibited random type spatial pattern [43]. Although CCR of *C. stenophylla* was greater than CSR in the semi-arid zone, their difference was relatively smaller (Table 4). Moreover, the advantage of clonal reproduction is that it is less affected by the environment changes and disturbances, and thus has higher in success potential in stressful environments. These might be the reason why *C. stenophylla* keeps a certain proportion of clonal reproduction in the semi-arid zone (32.7%, Table 4).

The CSRs of *C. stenophylla* were higher than CCRs in the arid zone (2.34 times) and substantially higher in the very arid zone (4.78 times). Clonal reproduction was a more economical reproduction method in areas with higher drought stress. Therefore, large portions of *C. stenophylla* offspring were ramet-derived in the arid zone population (60.0%) and the very arid zone population (72.5%, Table 4). At the same time, *C. stenophylla* maintained a certain proportion of seed-derived offspring in the arid zone (40.0%) and very arid zone (27.5%). These seed-derived individuals likely increase genetic diversity, adaptive capacity, and dispersal potential.

The CSR of *C. stenophylla* was much higher than (89.17 times) CCR in the intensively arid zone. Sexual reproduction was very costly (538.57 g biomass/sapling) in this zone. The high energy investment required for to sexual reproduction likely made it nearly impossible for seed-derived offspring to increase population growth. Moreover, the reproductive allocation to sexual of *C. stenophylla* was also very low in the intensively arid zone [39]. Therefore, sexual reproduction was impossible to act as the main population recruitment mode. In fact, we found that 100 *C. stenophylla* shrubs reproduced merely 1.05 saplings per years in the intensively arid zone [42], which is insufficient for population growth of *C. stenophylla*. In contrast, clonal reproduction was relatively economical in the intensively arid zone (6.04 g biomass/ramet). Clonal reproduction may provide a more reliable fitness gain than sexual reproduction in the intensively arid zone, thus it plays a more critical role in *C. stenophylla* population growth in this zone. Population recruitment substantially realized by clonal reproduction (89.3%) in the intensively arid zone. Previous studies have also shown that under the harsh conditions of dryland and desert, sexual reproduction is relatively rare [20, 46], plants rely on clonal reproduction to make up for insufficient or missing of seed reproduction, and thereby maintaining population growth [23]. Our results suggested that plants could not afford the excessive cost of sexual reproduction in these harsh environments. *C. stenophylla* population in the intensively arid zone mainly recruited by clonal reproduction, which resulted in clumped type spatial patterns in this zone [43]. Spatially aggregating ramets could also restrict sexual reproduction by competing with seedlings and reducing inter-genet pollination [12], which would contribute to the increase in CSR.

As climatic drought stress increased from the semi-arid zone to the intensively arid zone, this increasingly stressful environment drove a shift in the reproductive mode of *C. stenophylla* from more sexual reproduction in the semi-arid zone to more clonal propagation in the intensively arid zone. This was indicated by the sharp increase in CSR combined with a slight decrease in CCR. The lower the cost of one reproductive mode is, the larger the proportion of that reproduction mode is. These reproductive strategies reflected the principle of economical resource investment under resource-limited conditions, which might be a mechanism by which *C. stenophylla* has strong adaptive ability and can distribute across a broad geographic range.

In summary, for this clonal plant, reproductive cost was the dominant factor determining the shift between sexual and clonal reproduction modes. The effect of climate on reproductive mode appears to be mediated by the reproductive cost of each mode. The climate affects reproductive cost, especially the cost of sexual reproduction, and then drives the dominant reproductive mode in the population. Under favorable climate, cost of sexual reproduction is low, thus plants tend to choose sexual reproduction. In contrast, under harsh climate, the cost of clonal reproduction is far less than sexual reproduction, so clonal reproduction has an advantage. This mechanism would also explain why sexual reproduction is relatively rare in dryland and desert areas [20, 46].

### Sexual reproduction holds priority between the two reproductive modes

Our results also suggested that reproductive mode selection of *C. stenophylla* was not entirely mediated by reproductive cost. Sexual reproduction is prior in the two reproductive modes, which is another important factor mediating *C. stenophylla* reproduction strategy. Priority of sexual reproduction gradually increased from the semi-arid zone to the intensively arid zone (Fig. 1). The priority of sexual reproduction in the intensively arid zone was more prominent than those in the other zones. Although sexual production is very costly and it is not the main population recruitment way in the intensively arid zone, *C. stenophylla* did not completely discard this reproductive mode and kept a small proportion of sexual production (10.7%). Maintaining a certain proportion of sexual production for a clonal plant is beneficial, even while accruing expenses of vegetative growth [47], because occasional seed production is very important for maintaining certain level of genetic diversity in populations and promoting overall fitness [40]. Plants with dominant clonal reproduction and minor sexual production have been also found in other studies, such as *Arisaema triphyllum* [19], *Fragaria vesca* [6], *Spartina alterniflora* [48], *Acacia carneorum* [49]. Our results confirmed that sexual and clonal reproduction was unequal in evolutionary selection, and sexual reproduction holds priority between the two reproductive modes. Clonal plants would pay relatively high reproductive cost for the evolutionary benefits of sexual reproduction.

Bengtsson and Ceplitis [50] suggested that when the two kinds of propagules are similar to each other, evolution would tend towards a state dominated by the one or the other reproductive mode. Our results showed that sexual and clonal reproduction coexists in *C. stenophylla* populations, and the balance between them could shift along the climatic aridity gradient. These indicated that *C. stenophylla* populations have a seed-clonal reproductive system, which have been also found in other clonal species [6, 19, 48, 49]. Our results suggested that plants have polymorphism in reproduction property. Organisms should maintain a polymorphism in some traits, but only if each trait has a selective advantage in specific circumstances [51]. For *C. stenophylla*, the selective advantage of sexual reproduction is the evolutionary benefits in a benign climatic environment, while the selective advantage of clonal reproduction is to save resources in harsh climatic environment. Polymorphism in reproduction is an important characteristic for plants adaptation to varying environmental conditions.

## Conclusions

Based on our results, we can draw following conclusions: (1) Effect of climatic aridity is stronger on the cost of sexual reproduction than on the cost of clonal reproduction of *C. stenophylla* populations. As climatic drought stress increased from the semi-arid zone to the intensively arid zone, the sharp increase in cost of sexual reproduction and slight decrease in cost of clonal reproduction led to a great change in relative balance between cost of sexual and clonal reproduction, and thus resulted in a shift of reproduction modes of *C. stenophylla* populations. (2) For clonal plants, sexual reproduction has higher priority over clonal propagation, and plants would pay relatively high reproductive cost for the evolutionary benefits of sexual reproduction. However, if sexual reproduction cannot maintain population growth at a reasonable cost, *C. stenophylla* would choose clonal reproduction, and the ratio between sexual and clonal reproduction could be mediated by reproductive cost.

Our results indicated that for a common clonal plant species in the Inner Mongolian Steppe, both environmental stressors that cause one mode to be more costly over another and priority effects that arise from evolutionary advantages of sexual reproduction are both at play simultaneously. The shifts between reproductive modes not only maintain evolutionary and expansion advantage, but also reflected the principle of optimal resource utilization.

## Abbreviations

CCR: Cost of clonal reproduction
CSR: Cost of sexual reproduction
SRPI: Sexual reproduction priority index
